# Factors influencing adoption intentions to use AIGC for health information: findings from SEM and fsQCA

**DOI:** 10.3389/fpubh.2025.1525879

**Published:** 2025-04-25

**Authors:** Jing Liu, Xiaohan Chen, Chengzhi Liu, Pu Han

**Affiliations:** School of Management, Nanjing University of Posts and Telecommunications, Nanjing, China

**Keywords:** AIGC, health information, adoption intention, UTAUT, health belief model

## Abstract

**Background:**

With the rapid advancement of artificial intelligence technologies, AI-generated content (AIGC) was increasingly applied in the health information sector, becoming a vital tool to enhance the efficiency and quality of health information exchange.

**Purpose:**

This research investigated the motivations behind users’ adoption with AIGC during health information searches, aiming to advance public health management and technological innovation in health information.

**Methods:**

The study employed a model constructed from the UTAUT and the Health Belief Model. Comprehensive analysis of survey data was conducted using Structural Equation Modeling (SEM) and Fuzzy-Set Qualitative Comparative Analysis (fsQCA). Data handling and model verification were performed using SPSS 27, SmartPLS 4, and fsQCA 4.1 software tools.

**Results:**

The SEM results reveal that performance expectancy, effort expectancy, perceived susceptibility, perceived severity, perceived benefits, perceived barriers, and self-efficacy significantly positively influence adoption intentions, while facilitating conditions showed no significant effect. Fuzzy-Set Qualitative Comparative Analysis identifies two pathways that trigger adoption intention: Comprehensive Support and Health-Dominated.

**Conclusion:**

The study integrates the UTAUT and HBM in the context of health information technology adoption intention and employs a hybrid approach to deepen understanding of user behavior in the health information environment. Further exploration of emerging theories suitable for the rapidly evolving field of health information technology is still needed.

## 1 Introduction

A governance framework for population health management is established by the 14th Five-Year Plan for National Health and the Healthy China 2030 Plan Outline, both of which are strategic national policy. The need for precise and up-to-date health information is growing as public health awareness increases. Health information plays a critical role in guiding the public to make informed health choices, promoting healthy behavior changes, and effectively responding to public health emergencies. However, the rapid development of information technology has led to an exponential increase in health information (1), giving the public more opportunities to access health advice but also causing issues such as information overload and varying quality (2). In this context, ensuring the quality and accuracy of health information has become a major concern (3).

Artificial Intelligence Generated Content (AIGC) is one of the key technologies to address the challenges posed by health information. By automatically generating, filtering, and optimizing information through intelligent algorithms, AIGC fundamentally changes the way information is acquired and disseminated (4), greatly improving the efficiency and personalization of health information services and providing strong support for public health decision-making. However, the application of AIGC also presents new challenges. The acceptance of health information generated by AIGC among the public is influenced by factors such as technological characteristics, content of information, personal beliefs, and social environment. These factors collectively determine the effectiveness of AIGC in health information services.

This study aims to explore the key factors influencing users’ adoption intentions of AIGC in the health information domain. From the perspectives of technology acceptance and health behavior, the study conducts an in-depth analysis of users’ behavioral motivations when faced with health information generated by AIGC. This research reveals how various factors affect the adoption intentions of AIGC health information, providing theoretical and practical guidance for enhancing the application effectiveness of AIGC technology in health information services. To this end, the core contribution of this study has two aspects:

- To construct a theoretical framework based on the Unified Theory of Acceptance and Use of Technology (UTAUT) and the Health Belief Model (HBM), that bridges technological adoption theories with health behavior mechanisms, specifically addressing AIGC’s dual challenges in healthcare content generation and user decision-making.
- To investigate the proposed framework by employing a mixed-method approach combining Structural Equation Modeling (SEM) and Fuzzy-Set Qualitative Comparative Analysis (fsQCA) that decodes non-linear adoption logics in AIGC health information.

The rest of this study is organized as follows: section 2 reviews the literature on health information adoption intentions and the role of AIGC in the health domain. Section 3 presents the theoretical models used in the study, discusses hypothesis development, and introduces the research model. Section 4 outlines the methodology, including data collection and survey instruments. Section 5 presents the results from SEM and fsQCA analysis. Section 6 discusses the findings and their implications. Section 7 concludes with key findings, practical recommendations, and future research directions.

## 2 Literature review

### 2.1 Health information

In recent years, research in the field of health information has mainly focused on two directions: the mechanisms of health information dissemination and acquisition, and the key factors influencing the adoption intentions of health information.

Health information refers to information related to physical and mental health, disease prevention, nutrition knowledge, and wellness practices (5), which significantly impacts people’s health behaviors (6). Traditionally, health information has been provided by healthcare professionals such as doctors and nurses (7). However, the rise of modern information technology has made blogs, social media, and online forums important sources of health information (8). Accurate and accessible health information can encourage the public to adopt scientifically sound health behaviors (9). Nevertheless, the proliferation of information sources, while providing convenience for users, has also led to issues such as unregulated information quality and the spread of misinformation, which can result in severe personal or societal health consequences (10). For example, during the COVID-19 pandemic, the internet was flooded with misinformation, conspiracy theories, and pseudoscientific remedies regarding the virus’s origin, diagnosis, treatment, prevention, and transmission (11), significantly hindering pandemic control efforts (12, 84). Therefore, users need to assess the credibility of health information to effectively filter out misleading information. Effective dissemination of health information can significantly improve public health literacy and encourage preventive health behaviors (13). Furthermore, studies have shown that participants in online health communities often experience a sense of community support (14), which enhances their confidence in managing personal health and promotes positive changes in health behaviors (15).

Factors influencing the adoption intentions of health information have also been a research focus. The research model proposed by Huang J. C. (16) indicates that perceived ease of use, perceived usefulness, perceived benefits, perceived disease threat, action barriers, and internal and external cues to action significantly influence user attitudes, which in turn affect adoption intentions. Noor et al. categorized the factors influencing health information adoption into push and pull factors based on internal and external motivations (17). Push factors include lack of information, reluctance to seek professional advice for minor illnesses, dissatisfaction with healthcare providers’ attitudes, and inconvenience in consulting. Pull factors, on the other hand, involve the integrity of doctors, consideration of optimal treatment options, high professional skills, and adherence to professional ethics. Lee et al. (18) identified differences in health information acquisition between online and offline environments. The online environment, with its high autonomy and wide access to information, influences users’ health information-seeking behaviors by allowing them to explore diverse health topics independently. In contrast, the offline environment, through professional guidance and personalized interactions from doctors, fulfills users’ needs for precise and specific medical advice.

Although research on health information is relatively extensive, there is still a lack of attention to the role of emerging information technology features, such as real-time updates, personalization, and interactivity, in health information adoption. Moreover, existing literature has not adequately considered the unique characteristics of health information, especially its high requirement for accuracy. Therefore, more comprehensive and interdisciplinary research is needed to provide a theoretical foundation for designing more effective health information services and to offer practical recommendations for improving public health.

### 2.2 Adoption intentions of information in the AIGC environment

Technological advancements have driven continuous updates and expansions in the study of adoption intentions for various types of information to adapt to new informational environments (19). In October 2022, the launch of ChatGPT sparked explosive growth in the AIGC industry, garnering widespread attention across various fields (20). AIGC has revolutionized the way users acquire information and can be categorized into three participation modes based on the degree of AI involvement: partial participation, collaborative participation, and full participation. These changes are particularly evident in the diversification of information generation entities, the automation of processes, and the enhancement of efficiency (21). In the AIGC environment, information behaviors can be classified into two types: non-task-driven and task-driven (22). Non-task-driven research typically does not involve specific information search goals, hence the content is relatively broad, covering topics such as the development history (23, 83), technological characteristics (24), development opportunities (25), challenges (26), and future strategies (27) of AIGC. In contrast, task-driven research primarily assists users in accomplishing specific information query tasks to meet particular informational needs, making it possible to conduct more in-depth analyses of factors influencing the adoption intentions of AIGC-generated information. For instance, in academic practices at the graduate level, performance expectancy, individual innovativeness, and effort expectancy significantly influence the adoption intentions of AIGC (28). In information search contexts, different factors play distinct roles at various stages. During the information seeking stage, user attitudes are influenced by technological characteristics and human-computer interaction; in the information selection stage, comparisons between human intelligence and AI-generated intelligence critically impact evaluation and decision-making; while in the information utilization stage, the characteristics of conversational search engines affect users’ attribution of service failures and their overall experience (29). In the healthcare sector, AIGC is considered a “potential ally” in healthcare services, where its perceived competence and the credibility of the information provided are crucial (30).

Despite the proven support and efficiency improvements offered by AIGC in many areas, research on its application in the health information field is still relatively scarce. This is not only due to the development demands of AIGC itself but also because of the unique characteristics of health information. Health information requires a high degree of accuracy and timeliness. Moreover, the need for personalized handling of health information, combined with the broad range of public health issues, imposes higher demands on AIGC. These include the ability to accurately understand and process complex health condition descriptions and to generate useful recommendations based on users’ specific health backgrounds.

## 3 Theoretical model and research hypotheses

### 3.1 Theoretical model

Understanding the motivations behind users’ acceptance of AIGC technology in the health information environment is the core focus of this research. The study utilizes two theoretical frameworks: The Unified Theory of Acceptance and Use of Technology (UTAUT) and the Health Belief Model (HBM) to analyze users’ adoption intentions toward health information provided by AIGC.

The UTAUT model was proposed by Venkatesh et al. in 2003 by integrating the strengths of eight existing models (31, 32). It serves as an effective tool for understanding users’ acceptance of new technologies and explains their technology acceptance and usage behaviors through four key variables: performance expectancy, effort expectancy, social influence, and facilitating conditions. UTAUT has been widely applied to study individuals’ acceptance of information systems. For example, Abbad (33) confirmed the utility of UTAUT in predicting students’ behavioral intentions and actual use of e-learning systems. Gu (34) used UTAUT to evaluate the acceptance of e-health technologies among residents of developing countries. Researchers have extended the applicability of UTAUT by modifying variables or combining it with other models, applying it to diverse fields such as WeChat Moments (35), NFT products (36), e-government (37), and shared mobility services (23). Related studies indicate that UTAUT performs well in analyzing users’ adoption intentions of new technologies. Therefore, this study incorporates UTAUT into the theoretical framework.

The Health Belief Model (HBM) is one of the classic theories in health behavior research, widely used to explore the motivations behind individuals’ engagement in various health-related behaviors. Rosenstock clarified the historical origins of the model in 1974, noting that it was developed by a group of researchers from the Public Health Service between the 1950s and 1960s while addressing a series of independent applied research questions (38). Traditionally, the HBM has been primarily used to explain health behaviors directly related to disease prevention and management, such as breast cancer screening (39) and medication adherence (40). However, as the understanding of health has expanded to a more holistic view, the model has been applied to a broader range of health-related behaviors (41), such as physical exercise (42), vaccination (43), and improvements in quality of life (44). These studies have demonstrated the effectiveness of the HBM in analyzing various health-related behaviors. Therefore, this study incorporates the HBM into the theoretical framework.

### 3.2 Research hypotheses

**Figure 1:**
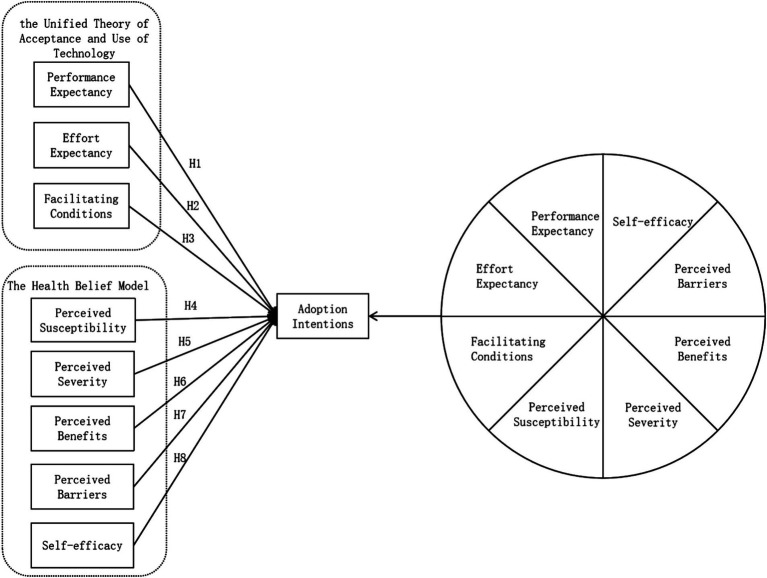
Research model.

The research model integrates performance expectancy (PE), effort expectancy (EE), and facilitating conditions (FC) from UTAUT, along with perceived susceptibility (PSu), perceived severity (PSe), perceived benefits (PBe), perceived barriers (PBa), and self-efficacy (SE) from the HBM, to propose a model for users’ adoption intentions (AI) of AIGC health information, as shown in Figure 1.

According to the tourism intention model, the following hypotheses are:

*H1*: Performance expectancy has a significant positive impact on adoption intentions.

*H2*: Effort expectancy has a significant positive impact on adoption intentions.

*H3*: Facilitating conditions have a significant positive impact on adoption intentions.

*H4*: Perceived susceptibility has a significant positive impact on adoption intentions.

*H5*: Perceived severity has a significant positive impact on adoption intentions.

*H6*: Perceived benefits have a significant positive impact on adoption intentions.

*H7*: Perceived barriers have a significant positive impact on adoption intentions.

*H8*: Self-efficacy has a significant positive impact on adoption intentions.

## 4 Methodology

### 4.1 Participants and data collection

This study gathered data from a varied set of people who were familiar with AIGC in the context of health information by means of an online survey. To guarantee they could comprehend the survey’s content and provide insightful answers, participants had to be at least 18 years old to meet the inclusion requirements.

A 450 questionnaires in all were collected. After a thorough screening procedure that eliminated incomplete surveys, replies completed in less than 60 s, or conflicting answers, 401 valid responses were kept, therefore producing an effective response rate of 89.11%. To guarantee a varied and representative sample for the study, the demographic data gathered included elements like age, gender, educational level, and medical history.

Data collection was carried out using the third-party online survey platform “Questionnaire Star”,^1^ which was independent of any institutional system. To ensure the confidentiality and anonymity of the participants, no personally identifiable information—such as names, email addresses, or phone numbers—was collected. Participants were informed about the purpose of the study and given an explanation of what AIGC in the health information context entailed, ensuring they fully understood the questionnaire and the focus on their adoption intentions toward AIGC-generated health information. Additionally, respondents were assured that participation was voluntary, and they could choose to withdraw from the survey at any time without any consequence. The data was collected over a period from April 26 to May 3, 2024. During this time, the project team ensured that all data was screened for quality and validity.

### 4.2 Instrument

The questionnaire consists of two sections: the first section collects basic information about the respondents, while the second section comprises the research scale, which is based on existing studies and adjusted for the specific context of AIGC and health information. The scale involves nine variables from the model, with each variable measured by three items. The questionnaire employs a 5-point Likert scale, where 1 represents “strongly disagree,” 3 represents “neutral,” and 5 represents “strongly agree.” The detailed items are listed in Table 1.

**Table 1:** Scale for factors influencing adoption intentions of health-related AIGC.

Variable	Item	Source
Performance expectancy	I can obtain the desired health information from health-related AIGC.	(28, 80)
	Using health-related AIGC effectively enhances my efficiency in acquiring health information.	
	The health information provided by health-related AIGC is accurate.	
Effort expectancy	The interface of health-related AIGC is simple and easy to operate.	(28, 80)
	The interface of health-related AIGC is simple and easy to operate.	
	I can easily understand the health information provided by health-related AIGC.	
Facilitating conditions	I have the necessary devices to use health-related AIGC.	(28, 80)
	My devices are compatible with the use of health-related AIGC.	
	My devices can connect to the internet smoothly to use health-related AIGC.	
Perceived susceptibility	People in today’s society often face various health issues.	(81, 82)
	Given my own health condition, I may experience some health problems within a year.	
	When I feel abnormal physical conditions, I might be facing a health issue.	
Perceived severity	Health problems have had a significant impact on my life or work.	(81, 82)
	Health problems have caused serious harm to my physical health.	
	Health problems have greatly affected my mental state or emotions.	
Perceived benefits	The health information provided by health-related AIGC helps me better understand my health issues.	(81, 82)
	The health information from health-related AIGC helps me prevent certain health problems.	
	The health information from health-related AIGC improves my quality of life.	
Perceived barriers	I believe that the health information obtained through search engines (e.g., Baidu) is not trustworthy.	(81, 82)
	I am not sure where to find reliable health information search resources.	
	Finding health information often consumes a lot of time and money.	
Self-Efficacy	I can effectively filter results when using search engines (e.g., Baidu) to obtain health information.	(82)
	My knowledge level is sufficient to support me in using health-related AIGC to search for health information.	
	I am confident in using health-related AIGC for self-health management.	
Adoption intention	I trust the health information provided by health-related AIGC.	(28, 80)
	I will prioritize using health-related AIGC to search for health information if I encounter health issues in the future.	
	I am willing to recommend the use of health-related AIGC to others.	

The development of the research scale was primarily based on established scales from the literature, ensuring that the measurement items were grounded in previous empirical studies. To tailor the scale to the specific context of AIGC and health information, some modifications to the wording of the items were made. These changes were intended to better reflect the unique aspects of AIGC technology in the health domain, while still maintaining fidelity to the original constructs and intent of the items. As a result, the revised scale ensures both theoretical robustness and contextual relevance for this study.

### 4.3 Data analysis

The data analysis was conducted using SPSS 27, SmartPLS 4, and fsQCA 4.1 software tools. First, SPSS 27 was employed to perform descriptive statistical analysis of the sample’s demographic characteristics, including age, gender, education level, and medical background. Next, Structural Equation Modeling (SEM) was carried out using SmartPLS 4 to test the research model and hypotheses. SEM helped examine the relationships between the constructs in the theoretical framework and assess both the direct and indirect effects on users’ adoption intentions toward AIGC-generated health information. Finally, Fuzzy-Set Qualitative Comparative Analysis (fsQCA 4.1) was utilized to explore causal relationships and identify distinct pathways leading to adoption intentions. fsQCA provided insights into the necessary and sufficient conditions for adoption, uncovering the complex interactions among factors that drive user behavior in the AIGC health information context.

### 4.4 Study variables

Performance expectancy (PE) refers to the user’s belief that using a particular technology will enhance their work performance. In the context of AIGC applications within the health information environment, performance expectancy involves the anticipated effectiveness of the technology in providing accurate health information (45), optimizing health management strategies (46), and improving health outcomes (47). Existing research has shown that users’ acceptance of health information technology is often directly related to their expectations regarding the technology’s ability to improve health outcomes (48). In the context of health information search, performance expectancy translates into users’ expectations of enhancing their ability to manage their health conditions through the use of technology (49).

Effort Expectancy (EE) refers to the perceived ease of learning and using a new technology. When users perceive AIGC as easy to use, they are more likely to adopt and utilize this technology. By automating complex data processing and providing intuitive user interfaces, AIGC has the potential to significantly reduce the effort required for users to search for and process health information, thereby enhancing their perceived ease of use. Research in the field of health information systems has shown that system usability significantly influences the acceptance of technology by healthcare professionals (50). Furthermore, effort expectancy plays a crucial role in users’ decisions to adopt new technologies, especially in health management applications that require frequent user interaction and data input, such as telemedicine services (51).

Facilitating Conditions (FC) refer to the degree to which users believe they have the necessary resources and support to adopt a specific technology. In the context of AIGC, facilitating conditions translate to the perceived external environment’s support for using AIGC, such as whether users have the required devices and network conditions to utilize AIGC and whether relevant providers offer sufficient compatibility support. The reliability and stability of the network are crucial for the effective functioning of e-health services (52). Additionally, some scholars have emphasized the importance of device compatibility for users’ acceptance of mobile health applications, suggesting that high compatibility between devices and applications can significantly enhance user experience and satisfaction (53). Especially in the context of health management, users often require immediate feedback and support to ensure the accuracy and practicality of health information. Therefore, perceived facilitating conditions are considered a critical predictor of the adoption of health information technology. This perception reduces the psychological and operational barriers potential users may face when using the technology, making them more inclined to accept AIGC to meet their health information needs.

Perceived Susceptibility (PSu) refers to an individual’s subjective assessment of their likelihood of developing a certain disease or health issue. It is used to explain how individuals’ perceptions of health risks influence their willingness to take preventive actions. Research has shown a significant positive correlation between high perceived susceptibility and individuals’ engagement in proactive health behaviors (54). When individuals believe they are at risk of a health threat, they are more likely to adopt health information and take action (55). In the design of health-related AIGC, considering perceived susceptibility can help enhance the level of personalized services. By adjusting the presentation of information to match the user’s perceived susceptibility level, it can improve the acceptance of information and the effectiveness of behavior change (56). When AIGC effectively identifies and provides personalized information targeting users’ specific health risks, individuals with high perceived susceptibility are more likely to value this information and may be more motivated to make positive changes in their health behaviors.

Perceived Severity (PSe) refers to an individual’s recognition and evaluation of the seriousness of the consequences associated with a particular health threat. If individuals believe that the potential consequences of a health issue are severe enough, they are more likely to take actions to avoid these outcomes (57). There is a significant association between perceived severity and individuals’ engagement in health behaviors; when people recognize the severity of a health threat, they are more inclined to follow medical advice or change their lifestyle habits (58). In the field of digital health communication, health information can motivate individuals to adopt health behaviors by enhancing their perception of the severity of disease outcomes (59). For example, health information disseminated through the media can effectively raise public awareness of the severity of specific health risks, thereby encouraging preventive behaviors (60). In the application of AIGC, more accurate and personalized health information can be generated to enhance users’ understanding of the serious consequences of diseases, thereby fostering a more proactive adoption intention toward health information.

Perceived Benefits (PBe) refer to an individual’s belief that engaging in a certain health behavior will result in positive health outcomes. Perceived benefits can significantly drive individuals’ health decisions and are a crucial factor in determining whether people take preventive measures (38). Further research has also confirmed the critical role of perceived benefits in promoting health behaviors, especially in disease prevention (58). In the context of health information, ensuring that users can clearly understand the specific health benefits of adopting a particular technology is key to increasing its adoption rate. For example, when searching for health information online, clearly presenting the potential benefits of health behaviors can effectively increase user engagement and adherence (61). The high level of personalization and precision offered by AIGC can provide significant advantages in health management, such as more accurate health monitoring and interventions, which can greatly enhance users’ perception of its potential health benefits. Moreover, the impact of perceived benefits is also reflected in the continued use of technology. Studies have shown that when users perceive that a health application can significantly improve their quality of life, they are more likely to continue using it (62). Similarly, if users believe that adopting AIGC can concretely improve their health management, for example, through personalized health advice, disease prevention information, or support for behavior change, they are more likely to accept and utilize this technology.

Perceived Barriers (PBa) are another core concept in the Health Belief Model, referring to the potential difficulties an individual perceives when considering whether to engage in a certain health behavior. Even if individuals recognize the potential benefits of a health behavior, they may still refrain from taking action due to perceived barriers (38). When using traditional methods to search for health information, individuals often encounter numerous challenges, such as time consumption, accessibility issues, information overload, and difficulties in assessing the reliability of the information. For example, information overload often leads to stress, which can negatively affect individuals’ willingness to seek and adopt health information (63). Similarly, the unreliability of information is one of the main obstacles preventing people from utilizing online health resources (64). In traditional health information acquisition, common barriers also include difficulties in understanding medical jargon and geographic and economic limitations in accessing professional health resources (65). However, in the design of AIGC health information systems, understanding these traditional barriers and taking measures to alleviate or eliminate them can significantly improve the system’s usability and user acceptance. It is important to note that the perceived barriers mentioned in this study refer to the difficulties individuals face when using traditional methods to gather health information, rather than those associated with using AIGC. For instance, AIGC systems can reduce the difficulty of use and address information overload issues by providing simplified interface designs, clear information guidance, and personalized information recommendations based on user behavior.

Self-efficacy (SE) refers to an individual’s confidence in their ability to successfully perform a specific health behavior. Social Cognitive Theory emphasizes that self-efficacy is a core factor influencing whether individuals will engage in health behaviors (66). In the context of AIGC, self-efficacy focuses on users’ confidence in their ability to use technology to acquire and apply health information. Individuals with high self-efficacy are more likely to adopt and maintain health behaviors because they believe they have the ability to control these behaviors. Enhancing self-efficacy can help individuals more effectively utilize health information and resources, thereby improving their ability to manage their own health (67). Self-efficacy has been positively correlated with health behavior changes, particularly in areas such as healthy eating and physical activity (68). Additionally, studies have shown that self-efficacy is a critical factor in determining whether individuals engage in HIV prevention behaviors (69).

## 5 Results

### 5.1 Sample characteristics

The results indicate a relatively balanced gender distribution, with male and female respondents accounting for 52.1 and 47.9%, respectively. Young individuals, with a wide range of educational backgrounds, constituted the main respondent group. Occupationally, medical students and professionals accounted for 16.7 and 19.2% respectively, thereby making 35.9% overall. This rather large percentage of respondents with a medical background helps the study to more professionally and thoroughly reflect the application effects of AIGC in the field of health information.

### 5.2 SEM results

#### 5.2.1 Evaluation of measurement models

SmartPLS 4 software was used for analysis of the survey results. Table 2 shows that the results of the reliability and validity analysis demonstrate that the Cronbach’s alpha (*α*) and Composite Reliability (CR) values for all measured variables are above 0.7, therefore implying great dependability and good internal consistency of the scale. Good convergent validity of the measuring scale is shown by the standardized factor loadings of all items exceeding 0.5 and the average variance extracted (AVE) for every latent variable being also larger than 0.5.

**Table 2:** Reliability and convergent validity analysis.

Variable	Measurement item	Factor loading	Cronbach’sα	CR	AVE
PE	PE1	0.816	0.824	0.893	0.737
	PE2	0.924			
	PE3	0.831			
EE	EE1	0.857	0.824	0.895	0.739
	EE2	0.868			
	EE3	0.854			
FC	FC1	0.862	0.720	0.840	0.637
	FC2	0.758			
	FC3	0.770			
PSu	PSu1	0.807	0.764	0.864	0.679
	PSu2	0.829			
	PSu3	0.837			
PSe	PSe1	0.862	0.834	0.901	0.752
	PSe2	0.903			
	PSe3	0.835			
PBe	PBe1	0.858	0.827	0.896	0.742
	PBe2	0.844			
	PBe3	0.882			
PBa	PBa1	0.850	0.799	0.882	0.713
	PBa2	0.846			
	PBa3	0.838			
SE	SE1	0.847	0.763	0.862	0.676
	SE2	0.831			
	SE3	0.788			
AI	AI1	0.851	0.791	0.878	0.705
	AI2	0.838			
	AI3	0.830			

As shown in Table 3, the AVE values for every latent variable have square roots that surpass their correlations with other factors, therefore demonstrating outstanding discriminant validity of the scale. It can be concluded that the measuring instruments applied in this study show great validity and dependability, therefore offering a strong basis for the subsequent structural model analysis.

**Table 3:** Discriminant validity analysis.

Variable	PE	EE	FC	PSu	PSe	PBe	PBa	SE	AI
PE	0.859								
EE	0.569	0.860							
FC	0.421	0.309	0.798						
PSu	0.569	0.408	0.288	0.824					
PSe	0.518	0.456	0.264	0.349	0.867				
PBe	0.478	0.401	0.269	0.393	0.422	0.861			
PBa	0.453	0.359	0.326	0.258	0.331	0.291	0.844		
SE	0.680	0.509	0.380	0.471	0.436	0.356	0.358	0.822	
AI	0.642	0.511	0.320	0.481	0.484	0.451	0.431	0.556	0.840

#### 5.2.2 Hypothesis testing

**Figure 2:**
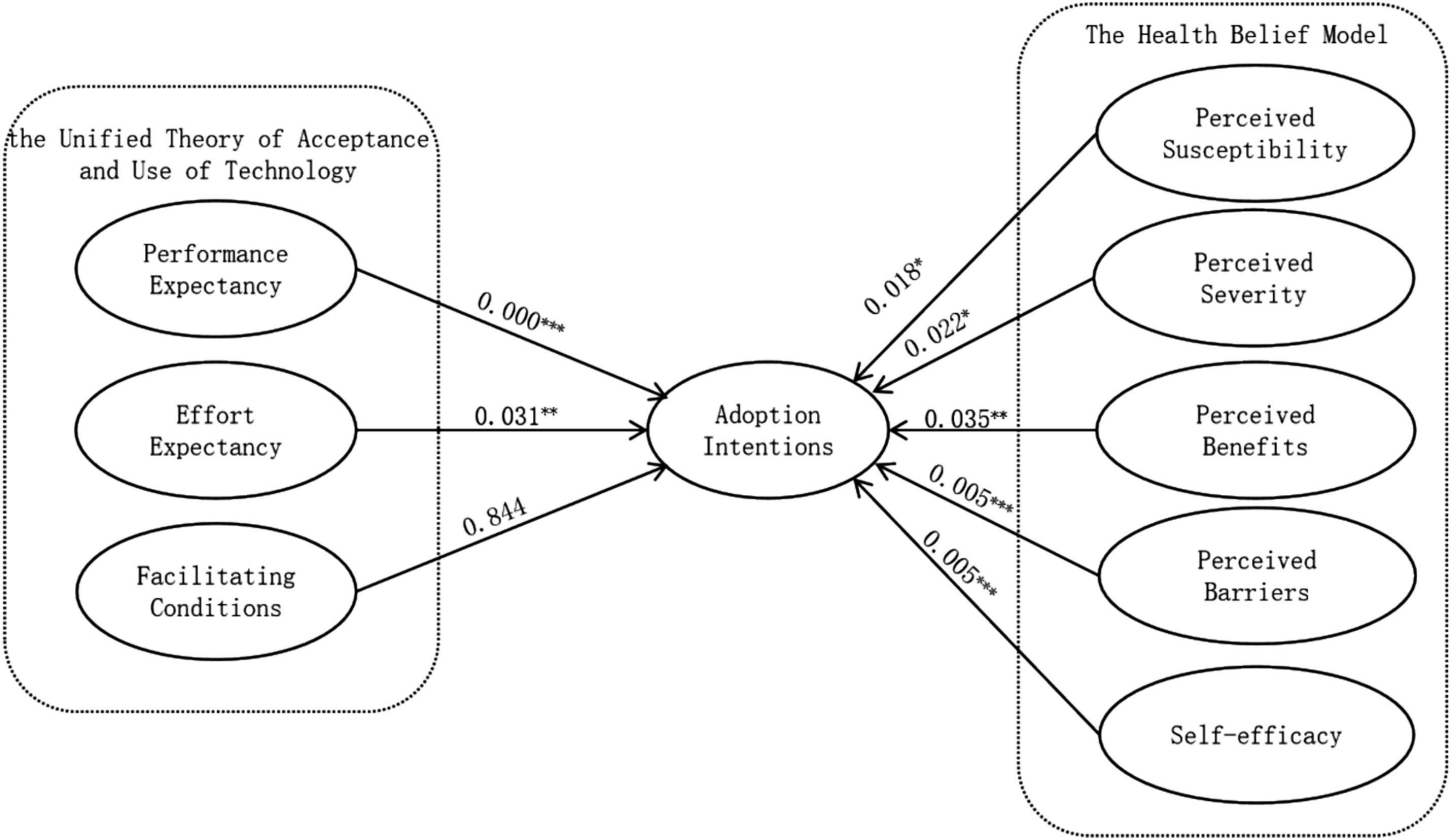
Model testing diagram.

As shown in the hypothesis testing results in Table 4, the path analysis indicates that, except for hypothesis H3 (*β* = −0.008, *p* = 0.844 > 0.05), which did not meet the statistical significance criterion, all other hypotheses were supported (*β* > 0, *p* < 0.05). This suggests that, with the exception of hypothesis H3, all the relationships in the model are statistically significant (Figure 2).

**Table 4:** Hypothesis testing analysis.

Hypothesis	Path	β	T-value	*p*-value	Conclusion
H1	PE → AI	0.252	4.008	0.000	Supported
H2	EE → AI	0.108	2.155	0.031	Supported
H3	FC → AI	−0.008	0.197	0.844	Not Supported
H4	PSu → AI	0.111	2.368	0.018	Supported
H5	PSe → AI	0.116	2.288	0.022	Supported
H6	PBe → AI	0.107	2.113	0.035	Supported
H7	PBa → AI	0.130	2.816	0.005	Supported
H8	SE → AI	0.145	2.799	0.005	Supported

### 5.3 fsQCA results

Numerous factors affect AIGC’s adoption intentions in the health information environment; these elements interact intricately under the multiple conjunctural causation. Testing single causal links using Structural Equation Modeling (SEM) by itself does not help one to fully explain this complexity. The study uses the Fuzzy-Set Qualitative Comparative Analysis (fsQCA) approach to obtain better understanding of these influencing mechanisms. This method complements the net effects investigated by SEM by analyzing antecedent variables from a configurational perspective, therefore investigating the mechanisms influencing the adoption intentions of health-related AIGC.

#### 5.3.1 Data calibration

The measuring variables have to be calibrated before doing qualitative comparative analysis. To guarantee the results are interpretable and practically relevant, the calibration method takes into account the categorical and degree variations among instances, thereby converting the original case data into set membership scores (70). With thresholds set at 95% for complete membership, 5% for full non-membership, and 50% as the crossover point (71), the 5-point Likert scale, for instance, is turned into a range from 0 to 1. In this work, values calibrated to 0.5 were gently changed to 0.501 in order to prevent the loss of case data. Table 5 shows some of the adjusted statistics.

**Table 5:** Data calibration results (partial).

PE	EE	FC	PSu	PSe	PBe	PBa	SE	AI
0.27	0.10	0.95	0.73	0.15	0.18	0.40	0.03	0.03
0.73	0.95	0.88	0.30	0.11	0.38	0.95	0.88	0.73
0.73	0.88	0.95	0.501	0.82	0.95	0.99	0.73	0.88
0.88	0.95	0.38	0.88	0.82	0.88	0.95	0.05	0.73
0.38	0.95	0.95	0.88	0.82	0.73	0.99	0.73	0.95
0.03	0.05	0.08	0.22	0.11	0.05	0.05	0.05	0.10
0.501	0.41	0.38	0.73	0.82	0.12	0.81	0.88	0.501
0.95	0.95	0.38	0.88	0.82	0.501	0.501	0.73	0.88
0.08	0.73	0.38	0.15	0.91	0.73	0.3	0.22	0.07
0.88	0.88	0.501	0.88	0.82	0.73	0.501	0.88	0.88
0.95	0.501	0.38	0.73	0.91	0.88	0.99	0.73	0.501
0.73	0.501	0.38	0.30	0.91	0.95	0.40	0.73	0.40
0.27	0.18	0.27	0.40	0.39	0.501	0.07	0.05	0.15
0.95	0.501	0.88	0.73	0.91	0.95	0.81	0.73	0.88
0.501	0.05	0.27	0.30	0.68	0.05	0.40	0.22	0.03
0.73	0.501	0.73	0.95	0.68	0.88	0.95	0.501	0.40
0.01	0.03	0.03	0.88	0.03	0.05	0.03	0.03	0.05
0.501	0.501	0.88	0.88	0.91	0.73	0.95	0.95	0.95
0.88	0.73	0.501	0.88	0.91	0.73	0.99	0.95	0.501
0.88	0.73	0.38	0.73	0.39	0.73	0.30	0.88	0.73

#### 5.3.2 Analysis of necessary conditions for single variables

According to the results of the necessity analysis presented in Table 6, the consistency scores for all antecedent variables are below 0.9 (72), indicating that no single variable constitutes a necessary condition for influencing adoption intentions.

**Table 6:** Results of necessary condition analysis.

Outcome variable: AI		
Condition variable	Consistency	Coverage
PE	0.825475	0.760860
~PE	0.508205	0.550249
EE	0.768811	0.758522
~EE	0.587844	0.590828
FC	0.756789	0.735686
~FC	0.585615	0.597670
PSu	0.779094	0.757833
~PSu	0.575878	0.587355
PSe	0.801453	0.735915
~PSe	0.520225	0.565796
PBe	0.783842	0.709219
~PBe	0.500401	0.553973
PBa	0.750514	0.744307
~PBa	0.562214	0.562117
SE	0.794866	0.770241
~SE	0.559759	0.573206

#### 5.3.3 Configuration analysis of conditions

Through configuration analysis of the data, 28 different causal combinations were identified. The consistency threshold was set at 0.8, the case threshold was set at 5, and the Proportional Reduction in Inconsistency (PRI) consistency threshold was set at 0.75, resulting in a truth table containing combinations that meet the threshold criteria (73). Based on these combinations, seven distinct configurations were ultimately generated, all with consistency values greater than 0.9, and a total consistency of 0.906, which exceeds the threshold of 0.8. This indicates a high level of consistency among these configurations, with approximately 91% of users displaying adoption intentions. The overall coverage was 0.599, which is greater than 0.5, indicating a high explanatory power (74). This suggests that these seven configurations explain approximately 60% of the reasons for the adoption intentions of health-related AIGC. From these results, two configuration paths were identified, providing important insights for further analysis and research (Table 7).

**Table 7:** Results of condition configurations.

Antecedent variables	Comprehensive support path						Health-dominated path
Configuration	A	B	C	D	E	F	G
PE	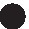	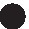	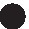	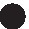	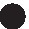	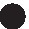	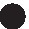
EE	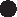			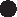	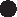	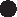	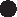
FC	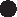	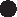	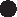	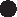	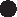		⊗
PSu		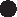		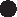	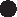	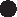	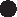
PSe	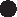	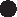	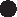	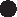		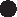	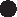
PBe	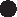	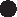	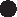		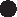	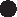	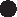
PBa	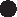		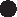	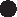	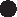	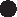	⊗
SE		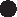	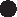	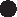	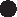	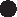	⊗
Raw coverage	0.413	0.434	0.412	0.396	0.382	0.393	0.227
Unique coverage	0.029	0.037	0.008	0.037	0.023	0.022	0.010
Consistency	0.937	0.950	0.943	0.945	0.942	0.945	0.956
Overall coverage	0.599						
Overall consistency	0.906						

“
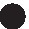
” and “
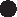
” indicate the presence of the condition variable, while “⊗” indicates the absence of the condition variable. A blank space indicates that the condition variable is not relevant (it can be either present or absent). “
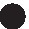
” represents a core condition, and “
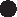
” represents a peripheral condition.

Based on the analysis, the study identified two configuration paths:

(1) Comprehensive Support Path

This path includes configurations A to F. Each of these configurations comprehensively addresses multiple dimensions that influence the adoption intentions of health-related AIGC, including performance expectancy, effort expectancy, facilitating conditions, perceived susceptibility, perceived severity, perceived benefits, perceived barriers, and self-efficacy. In these configurations, multiple factors jointly impact users’ adoption decisions, illustrating a complex and comprehensive decision-making environment.

In this model, performance expectancy is identified as the core condition, indicating that respondents generally consider the performance improvement brought by AIGC as the primary driving force for adopting this technology. This highlights the critical role of performance expectancy in motivating users to adopt health-related AIGC, providing strong support for hypothesis H1 in the study. Although performance expectancy is the core driving factor, other factors also play indispensable roles as supplementary conditions.

(2) Health-Dominated Path

This path consists of only one configuration, G, where performance expectancy is also the core condition. The peripheral conditions include effort expectancy, perceived susceptibility, perceived severity, and perceived benefits. However, facilitating conditions, perceived barriers, and self-efficacy are absent in this model.

This configuration shows that even though respondents might face potential usage barriers, such as insufficient facilitating conditions or uncertainty about their self-efficacy, they still place a high value on the performance improvements offered by AIGC. This indicates that respondents believe the quality and benefits of health information obtained through AIGC are sufficient to outweigh any existing practical or perceived barriers. In other words, performance expectancy plays a decisive role in driving their adoption of health-related AIGC. In this case, it can be inferred that respondents’ expectations of AIGC are entirely based on its potential to improve health outcomes. This suggests that users may still choose to adopt new technologies, even when their personal capabilities are insufficient, as long as they believe the technology can significantly enhance their health status.

#### 5.3.4 Robustness analysis

The study conducted robustness tests by adjusting the consistency level (from 0.8 to 0.85) and the calibration thresholds (replacing 95% with 90% for full membership and 5% with 10% for full non-membership) and by modifying calibrated values of 0.5 to 0.499. During the process of constructing the truth table and performing standardized analysis, it was found that the combination of configuration path factors remained largely consistent with the study’s original results, with only minor differences observed between the indicators. This indicates that the robustness and reliability of the analysis results have been verified, providing strong support for the interpretation and inference of the study’s conclusions.

## 6 Discussion

This study aimed to explore the factors influencing the adoption intentions of AIGC-generated health information. By employing Structural Equation Modeling (SEM) and Fuzzy-Set Qualitative Comparative Analysis (fsQCA), the study uncovered several key insights into the psychological and contextual drivers of technology adoption in health information. This section discusses the implications of these findings in the context of the research hypotheses, their alignment with previous literature, and the broader theoretical contributions.

### 6.1 Interpretation of SEM results

The SEM results support most of the hypothesized relationships, with performance expectancy, effort expectancy, perceived susceptibility, perceived severity, perceived benefits, perceived barrier and self-efficacy showing significant positive impacts on users’ adoption intentions of AIGC-generated health information. SEM path analysis indicates that performance expectancy has a significant positive impact on adoption intentions. This suggests that when AIGC can provide the health information users need, they are more willing to adopt it, as it aligns with their usage objectives, consistent with previous studies (48). Effort expectancy also has a significant positive impact on adoption intentions, indicating that users are more likely to adopt AIGC when they do not feel overburdened during the use process. User-friendly, easy-to-operate, and easy-to-understand AIGC products are more likely to be accepted by users, in line with prior research (50). Perceived susceptibility has a significant positive impact on adoption intentions, as the awareness of being at risk for diseases can enhance users’ preventive health consciousness. When users perceive a high risk of illness, they are more likely to adopt health-related AIGC as a tool for prevention and monitoring, aiming to detect potential health issues early and take action, consistent with previous research (55). Perceived severity also has a significant positive impact on adoption intentions. Users’ perception of the severity of disease outcomes drives them to rely on health-related AIGC for continuous monitoring and management, to reduce the severe consequences of diseases. This reliance helps increase the frequency of AIGC use and user engagement, which is in line with earlier studies (57, 58). Perceived benefits have a significant positive impact on adoption intentions. If health-related AIGC can provide personalized health advice, accurate diagnosis, or effective treatment options, users will find using AIGC worthwhile, thus increasing their adoption intentions, consistent with previous research (62). Perceived barriers to traditional health information seeking have a significant positive impact on adoption intentions. AIGC has effectively addressed issues associated with traditional health information methods, such as information overload, accessibility, and reliability issues, reducing the difficulty for users in finding health information and making them more willing to use it, in line with earlier studies (63, 65). Self-efficacy has a significant positive impact on adoption intentions. The ease of use of AIGC enhances users’ confidence, encouraging them to use it more proactively, consistent with previous research (67).

Contrary to the UTAUT model’s expectations, however, facilitating conditions had no appreciable impact, implying a health specific contradiction whereby infrastructure preparedness loses importance to perceived health need. When compared with earlier investigations (53), this difference is very remarkable. The results imply two mutually reinforcing interpretations based on the high stakes character of health information environments. On one hand, consumers’ behavioral calculus favors performance expectancy over facilitating conditions when they confront imminent health threats such cancer. This trend reflects healthcare crises in which patients avoid logistical obstacles to seek distant specialist treatment (75), a phenomena essentially entrenched in the risk-as-feelings paradigm (76). Here, analytical appraisals of technology infrastructure are subordinated to emotional concerns of misdiagnosis or treatment delays. On the other hand, the digital literacy of modern people has normalized device compatibility, so transforming facilitating conditions from a differentiating factor into an assumed baseline (77), unlike low-resource environments where facilitating conditions remain fundamental (78). Together, risk-induced credibility prioritizing and digital normalizing rearrange adoption drivers, thereby separating health urgency from behavioral goals to separate infrastructure issues from ethical considerations. Users thus assess AIGC from a health need perspective: 24/7 availability, diagnostic accuracy, and tailored risk insights exceed technical convenience, implying that UTAUT’s original assumptions (79) need contextual recalibration for high-stakes health technologies.

### 6.2 fsQCA findings and pathways

The fsQCA analysis provided valuable insights into the complex and conditional nature of adoption intentions for AIGC health information. The results indicated that there are no necessary conditions for adoption intentions. This suggests that no single factor is typically sufficient to determine high adoption intentions on its own. Instead, adoption intentions are the result of the interplay between multiple factors, which work together in different combinations to influence user behavior.

Despite the absence of necessary conditions, performance expectancy emerged as a core condition in all seven configurations, underlining its crucial role in shaping adoption intentions. This consistent presence of performance expectancy in all configurations highlights its centrality in influencing users’ decisions to adopt health-related AIGC. Regardless of the other factors involved, users’ belief in the effectiveness of AIGC in improving health outcomes remains a key driver of adoption. This finding reinforces the idea that the perceived usefulness of technology is a primary motivator in health technology adoption.

From the configuration analysis, two distinct pathways were identified: Comprehensive Support Path and Health-Dominated Path. Each representing a different combination of conditions leading to adoption intentions.

In the Comprehensive Support Path, almost every variable was present as a condition, suggesting that adoption intentions are influenced by a broad range of factors. In this path, performance expectancy remained the central driving factor, but other variables such as perceived benefits and self-efficacy also played significant roles. This pathway indicates that, to facilitate widespread adoption, AIGC technologies must not only demonstrate clear health benefits but also address concerns related to users’ ability to use the technology effectively. This aligns with findings in the literature that emphasize the importance of both the perceived utility of a technology and users’ confidence in their ability to engage with it. The Comprehensive Support Path has the highest explanatory power, as it accounts for a larger portion of the variability in adoption intentions.

In contrast, the Health-Dominated Path suggests that adoption intentions can still be high based on performance expectancy alone, even in the absence of other factors like facilitating conditions and self-efficacy. This pathway demonstrates that users may prioritize the health benefits of AIGC technology above all else. Even without strong technical support or confidence in using the technology, the perceived health improvements offered by AIGC can be sufficient to drive adoption. This highlights that the potential health benefits of AIGC technologies can outweigh perceived barriers related to ease of use or technical convenience, supporting the idea that health-related technology adoption is heavily influenced by the expected improvement in health outcomes.

### 6.3 Comparison of SEM and fsQCA results

Both approaches agree in stressing the unmatched relevance of performance expectancy. SEM ranks Performance Expectancy as the best predictor. FsQCA increases performance expectancy even further to a required condition in both paths, where its absence nullifies adoption independent of other considerations. This dual method validation shows, with great design ramifications, that users finally give performance expectancy top priority above peripheral characteristics.

The nonlinearity of perceived barriers is critical. While SEM paradoxically indicates a positive effect of perceived barriers, contradicting health behavior theories, this arises because users interpret mild residual obstacles as evidence of AIGC’s competence. However, SEM’s linear model masks perceived barriers’ directional reversal, conflating its dual roles into a net positive average. fsQCA resolves this contradiction: in Health-Dominated Path, perceived barriers’ strict exclusion reveals adoption collapses once barriers exceed critical thresholds, while in Comprehensive Support Path, perceived barriers’ irrelevance or marginal tolerance confirms low-intensity barriers are neutralized by performance expectancy. This threshold logic redefines perceived barriers as a situational sensitive critical regulatory factor rather than a static determinant. Similarly, self-efficacy role shifts from a skill amplifier in tech driven scenarios to an irrelevant factor in health crises, underscoring the necessity of pathway-specific interventions.

Though statistically insignificant in SEM, facilitating conditions show contextual asymmetry in fsQCA. Facilitating conditions serves as a resource amplifier in Comprehensive Support Path. On the other hand, facilitating conditions’ inactivity in Health-Dominated Path shows a motivating override mechanism. Users avoid infrastructure requirements to give rapid solutions top priority when health concerns rule. This dualism places facilitating conditions not as a supplement for performance expectancy but rather as a contingent facilitator whose worth only shows up in the adoption context allowing resource-sensitive assessment.

## 7 Conclusion

This study, based on the UTAUT and Health Belief Model, uses Structural Equation Modeling (SEM) and Fuzzy-Set Qualitative Comparative Analysis (fsQCA) to explore the interaction effects and mechanisms among various influencing factors. It examines the net effects driving users’ adoption of AIGC for health information and identifies the configurations that lead to adoption intentions. The study also addresses the limitations of SEM in handling variable interdependencies and causal analysis by using fsQCA to explore the joint effects of different factors.

### 7.1 Research conclusion

This study extends the application of UTAUT and Health Belief Model to the field of AIGC in health information, enhancing the theoretical understanding of the factors influencing adoption intentions. By employing a mixed-method approach, it provides deeper insights into the complex interactions among various antecedents and enriches our understanding of health information and AIGC adoption. The results confirm that performance expectancy is a key driver in users’ adoption and continued use of AIGC-generated health information.

The study also highlights the critical role of AIGC in health information services, particularly in improving information acquisition efficiency, enhancing information credibility, and supporting personalized health management. These findings offer both theoretical and practical guidance for the development of more intelligent, user-friendly health information systems based on AIGC technologies.

### 7.2 Implications and limitations

This study provides several key implications for the development and implementation of AIGC technologies in health information.

- For users, educational portals on the “Healthy China” platform should let AIGC results be cross-verified against national medical standards. Community seminars might teach participants to give credibility first priority in high-risk situations—such as cancer screening.
- For developers, they especially need to incorporate the Disease AI Guidelines of authoritative medical institutions. Priority should be given multi-model validation and guideline-aligned transparency such as Chinese Medical Association citations.
- For healthcare practitioners, electronic health records must include evidence-based risk matrices and patient-specific biomarkers.
- For legislators, tiered rules should require blockchain audits for important AIGC uses thereby guaranteeing algorithm transparency under national medical device standards.

This study does have some limitations:

- Social desirability bias could have affected the questionnaire survey’s outcomes. Future studies may combine qualitative and quantitative approaches to provide a more all-encompassing view.
- The study mostly depended on accepted theoretical models like UTAUT and the Health Belief Model, which might not completely reflect the complexity of developing technologies like AIGC. New, developing theoretical models more suitable to grasp the fast changing field of health information technology should be investigated in future work.
- This work’s cross-sectional approach restricts the capacity to clarify the temporal processes behind AIGC health information uptake. Future studies should combine behavioral log analysis to find stage-specific causes with longitudinal monitoring to track users over the whole adoption lifespan, from first exposure to steady usage.

## Data Availability

The raw data supporting the conclusions of this article will be made available by the authors, without undue reservation.

## Footnotes

^1^ www.wjx.cn
